# A Case of Ovarian Metastasis of Pancreatic Cancer Causing Ovarian Torsion

**DOI:** 10.7759/cureus.21352

**Published:** 2022-01-17

**Authors:** Shohei Tanabe, Sachiyo Sugino, Kotaro Ichida, Kiyoshi Niiya, Syuji Morishima

**Affiliations:** 1 Obstetrics and Gynecology, Kobe City Medical Center West Hospital, Kobe, JPN

**Keywords:** laparoscopy, pancreatic cancer, ovarian cancer, metastases, torsion

## Abstract

A 55-year-old female who had been diagnosed with stage 4 pancreatic cancer visited the emergency department of our hospital owing to sudden lower abdominal pain and was found to have an ovarian tumor. An urgent laparoscopic resection of the right adnexa was performed with a diagnosis of ovarian tumor stalk torsion. Postoperative pathological diagnosis revealed ovarian metastasis of pancreatic cancer. Our case report demonstrates that an ovarian tumor on one side in a patient with advanced pancreatic cancer may represent ovarian metastasis of the primary tumor.

## Introduction

Pancreatic cancer rarely metastasizes to the ovaries [1]. It is also rare for metastatic ovarian cancers to cause ovarian torsion [2]. In this case report, we report the first case of a metastatic ovarian tumor of pancreatic cancer resulting in ovarian torsion.

## Case presentation

A 55-year-old female visited the Department of Gastroenterology at our hospital with complaints of abdominal pain, nausea, and weight loss. She was diagnosed with pancreatic cancer (tubular adenocarcinoma) as a result of endoscopic ultrasound-guided fine needle aspiration. The computed tomography (CT) performed at that time did not show any ovarian tumor. After diagnosis, conversion surgery was attempted, but peritoneal dissemination was observed, and the patient was diagnosed as stage 4. Conversion surgery was canceled, and duodenal bypass surgery was performed, followed by anticancer drug therapy (gemcitabine and nab-paclitaxel).

**Figure 1:**
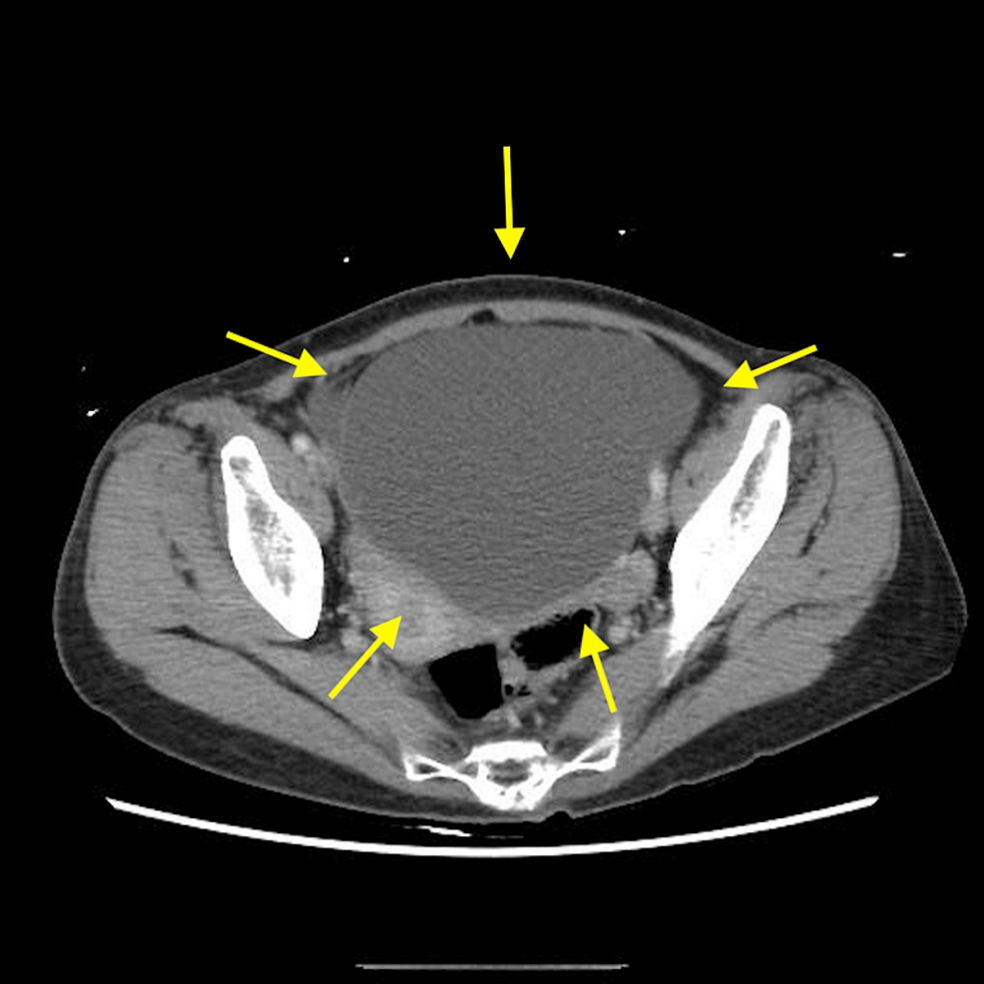
Computed tomography (CT) of the ovarian tumor Abdominal computed tomography showing a 10-cm-large monoblastic ovarian tumor Yellow arrows: ovarian tumor

**Figure 2:**
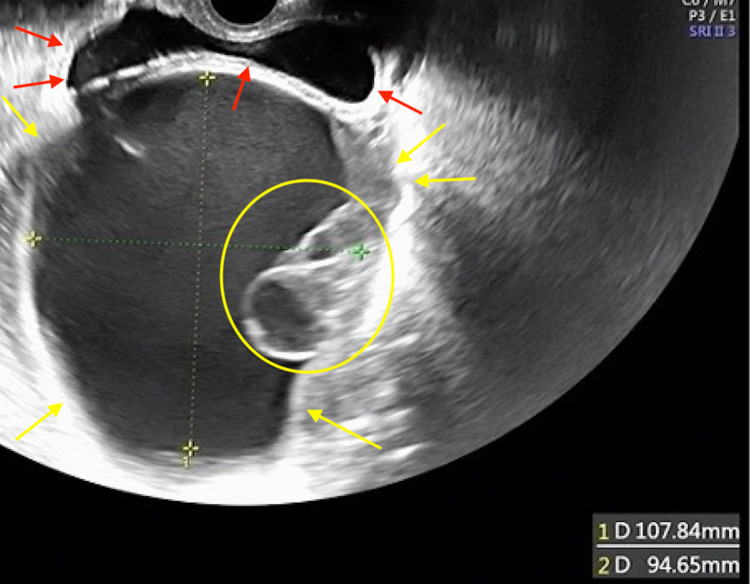
Transvaginal ultrasound image of the ovarian tumor Transvaginal ultrasound showing a 10-cm-large monoblastic ovarian tumor Yellow arrows: ovarian tumor; yellow circle: cystic component; red arrows: cyst

**Figure 3:**
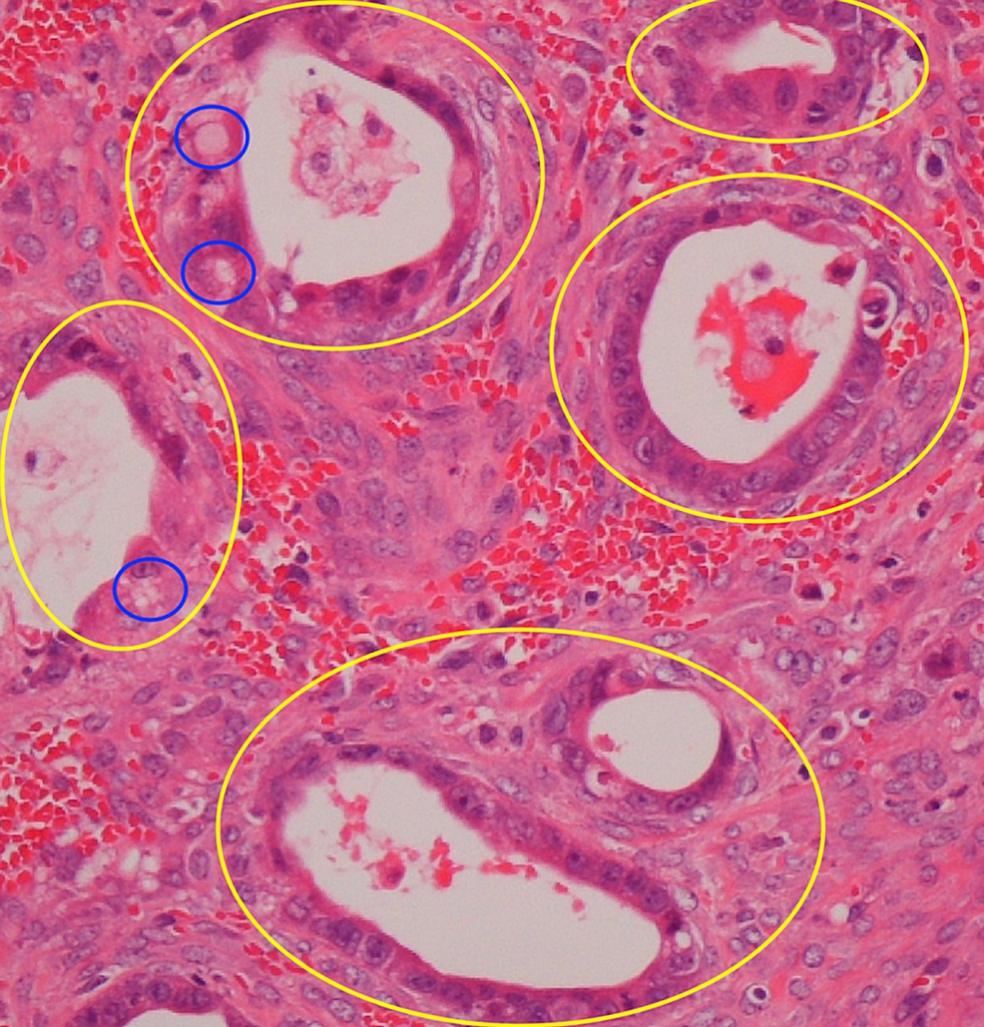
Hematoxylin and eosin stain (×200) Tumor cells with strong nuclear atypia growing with some glandular duct structures Yellow circles: glandular duct structure; blue circles: containing mucus component

Four months after the surgery, the patient visited our emergency room due to sudden abdominal pain. A CT scan revealed a 10-cm, thin-walled, smooth tumor in the pelvis (Figure 1). Transvaginal ultrasound revealed a 10-cm ovarian tumor with partial septa (Figure 2). Since ovarian torsion was suspected, an emergency laparoscopic resection of the right adnexa was performed at the Department of Obstetrics and Gynecology of our hospital. The intraoperative findings revealed that the right ovarian tumor had torsion. The left ovary was normal. The postoperative course was favorable, and the patient was discharged on the eighth postoperative day. The postoperative pathological diagnosis revealed adenocarcinoma with mucus production in the ovarian tumor (Figure 3). Although mucinous carcinoma was the differential diagnosis, we diagnosed cancer as ovarian metastasis of pancreatic cancer because the findings were similar to those of pancreatic cancer biopsied previously. After the surgery, the patient was treated with anticancer drugs at the Department of Gastroenterology of our hospital; the patient has shown no recurrence since one year following the surgery.

## Discussion

Ovarian metastases from non-gynecological organs, especially primary tumors of the digestive organs, are called Krukenberg tumors [3]. The pathological findings are similar to those of ovarian primary mucinous carcinoma; however, bilateral ovarian tumors are typical findings of Krukenberg tumors [4].

In this case, pancreatic cancer had metastasized to the ovaries, and a diagnosis of the Krukenberg tumor was formulated. However, we did not consider the possibility of a Krukenberg tumor when emergency surgery was performed for the following reasons.

First, pancreatic cancer is the primary tumor of metastatic ovarian cancer in only 3.3% of cases [3]. Second, in this case, the patient had only a right ovarian tumor, and the left ovary had normal findings. Third, few cases of Krukenberg tumor causing stalk torsion have been reported [5]. Fourth, in the typical CT findings of the Krukenberg tumor, the cystic and solid components are often mixed in the ovarian tumor [6], but in this case, only the cystic component was present.

These four points are atypical for Krukenberg tumors. Therefore, we first considered the possibility of a benign ovarian tumor, which is the most common cause of ovarian torsion.

It has been reported that it is difficult to differentiate mucinous ovarian cancer from metastatic ovarian cancer pathologically [6]. However, the ultrasound findings of Krukenberg tumors are characterized by a smooth tumor surface with cystic components inside, which is different from primary ovarian cancer with solid components and papillary projections inside the tumor [1]. In the present case, the ultrasound findings showed a smooth surface tumor with cystic components typical of a Krukenberg tumor. The specificity of the ultrasound findings is reportedly 96.5% [6], which was considered a characteristic finding of the Krukenberg tumor in this case [6].

Here, we discuss the effects of this surgery. Considering minimally invasive surgery for ovarian cancer, laparoscopic surgery for early-stage ovarian cancer requires careful attention owing to the risk of mortality due to membrane failure [7]. However, diagnostic laparoscopy has been reported to be effective in advanced ovarian cancer in predicting tumor progression and resectable areas [8]. Therefore, in cases with distant metastasis, such as in the present case, the failure of the tumor content during tumor removal may not have affected the patient's mortality. However, the surgical content should be considered.

It has been reported that cytoreductive surgery may be effective for metastatic ovarian cancer in patients with good performance status, metastases confined to the ovary, and residual disease less than 2 cm. Contralateral ovarian resection is reportedly necessary even if the tumor is unilateral [9]. Therefore, in this case, the contralateral ovary should have been resected to prevent recurrence in the future. Although no tumor was found in the left ovary at the time of follow-up, we plan to continue monitoring the patient for recurrence.

## Conclusions

In patients with advanced gastrointestinal cancers, previously unrecognized unilateral ovarian tumors may be ovarian metastases of the primary tumor. Ultrasound findings may show Krukenberg tumor findings even when CT findings do not raise suspicion of malignancy. If there is a possibility of the Krukenberg tumor, bilateral oophorectomy should be considered even if the tumor is found in only one ovary.
